# Evidence that infant and early childhood developmental impairments are associated with hallucinatory experiences: results from a large, population-based cohort study

**DOI:** 10.1017/S0033291721003883

**Published:** 2023-04

**Authors:** Eleanor Carey, Colm Healy, Yael Perry, Diane Gillan, Andrew J. O. Whitehouse, Mary Cannon, Ashleigh Lin

**Affiliations:** 1 Department of Psychiatry, Royal College of Surgeons in Ireland, Dublin, Ireland; 2 Trinity College Institute of Neuroscience, Dublin, Ireland; 3 Telethon Kids Institute, The University of Western Australia, Perth, WA, Australia; 4 Department of Psychology, Beaumont Hospital, Dublin, Ireland; 5 Department of Psychiatry, Beaumont Hospital, Dublin, Ireland

**Keywords:** Cognition, Early childhood development, Hallucinatory experiences, Motor

## Abstract

**Background:**

Cognitive and motor dysfunction are hallmark features of the psychosis continuum, and have been detected during late childhood and adolescence in youth who report psychotic experiences (PE). However, previous investigations have not explored infancy and early childhood development. It remains unclear whether such deficits emerge much earlier in life, and whether they are associated with psychotic, specifically hallucinatory, experiences (HE).

**Methods:**

This study included data from Gen2 participants of The Raine Study (*n* = 1101), a population-based longitudinal cohort study in Western Australia. Five areas of childhood development comprising: communication; fine motor; gross motor; adaptive (problem-solving); and personal-social skills, were assessed serially at ages 1, 2 and 3 years. Information on HE, depression and anxiety at ages 10, 14 and 17 years was obtained. HE were further subdivided into those with transient or recurrent experiences. Mixed effects logistic regression models and cumulative risk analyses based on multiple domain delays were performed.

**Results:**

Early poorer development in multiple areas was noted from ages 1, 2 and 3 years among youth who reported HE. Early developmental delays significantly increased the risk for later HE. This association was particularly marked in the recurrent HE group, with over 40% having early developmental delays in multiple domains. There was no significant association between early childhood development and later anxiety/depression apart from lower gross motor scores at age 3.

**Conclusions:**

The findings suggest that early pan-developmental deficits are associated with later HE, with the effect strongest for young people who report recurrent HE throughout childhood and adolescence.

## Introduction

Poor mental health is being reported at increasingly younger ages, and there has been much focus traditionally on affective disorders and anxiety (Caspi et al., 2020; Merikangas et al., 2010). However, there is growing recognition of the importance of psychotic phenomena in youth. Psychotic experiences (PE), the subclinical expressions of psychotic symptoms, occur in the absence of a psychotic disorder and mainly concern auditory (both verbal and non-verbal) and, less commonly, visual hallucinatory experiences (HE) (Kelleher, Harley, Murtagh, & Cannon, 2011). PE are most prevalent during late childhood, with 17% of 9–12 year olds within the general population reporting these phenomena (Kelleher et al., 2012a), and a further 12% of children and adolescents reporting auditory hallucinations specifically (Maijer, Begemann, Palmen, Leucht, & Sommer, 2018). A growing body of research suggests that PE are not only homotypically or inevitably linked to psychotic disorders (Healy et al., 2019), but also represent markers of vulnerability for a range of psychopathology and poorer functional outcomes into adulthood (Carey et al., 2021). PE have also been shown to be associated with suicidality and substance abuse in young people (Cederlöf et al., 2017; Honings, Drukker, Groen, & van Os, 2016; Kelleher, Cederlöf, & Lichtenstein, 2014). For the majority of young people (75–90%), PE are transient in nature (Calkins et al., 2017), but, in those for whom PE persist, greater risk of adverse outcomes have been shown (Calkins et al., 2017). The recurrence of PE therefore may be indicative of a more serious underlying psychopathological process.

Early childhood development, which includes physical, social and cognitive development, strongly influences mental health and well-being throughout the lifespan and is critical for influencing a child's developmental trajectory (Irwin, Siddiqi, & Hertzman, 2007). Early childhood represents a critical time for the early detection of risks for later psychopathology (Green et al., 2019). Cognitive variances in early life lead to a very high risk of not only developmental issues for children, but also social-emotional and behavioural problems at later ages (Carter, Briggs-Gowan, & Davis, 2004), and should be signalled for clinical intervention.

Investigations of early childhood development and its role in predicting later schizophrenia have been carried out using birth cohorts and data linkage approaches but less research has been carried out with HE as the outcome. Motor deficits during development likely represent an endophenotype for schizophrenia (Burton et al., 2016). Clarke et al. (2011), in a study analysing data from the Finnish population register, found that delayed milestones in infancy significantly increased the risk of a later diagnosis of schizophrenia, in a dose–response manner. Cannon et al. (2002), in analyses from a New Zealand birth cohort study, found that subtle but significant impairments in neuromotor, language and cognitive development were detectable from age 3 in children who reported psychotic symptoms at age 11 and were also detectable in individuals later diagnosed with schizophreniform disorder (but not depression, anxiety or mania) at age 26. However, developmental data were not available before the age of 3. Hameed et al. (2018) showed that a decline in early social and communication skills over the first 4 years of life, and a declining pattern of fine motor skills in males, was predictive of later PE (at age 12 years) in a UK birth cohort.

Most studies in this area have focused on childhood and adolescence, with research showing that cognitive and motor dysfunction at this age is also associated with PE (Carey et al., 2019, 2021; Mollon et al., 2016). A lag in cognitive development from age 8 years has been detected in children who later report PE (Gur et al., 2014). However, whether differences in developmental trajectory can be noted from infancy remains unclear. Questions pertaining to whether there is an association between early childhood developmental delays and poorer outcomes also remain.

Using data from Gen1 and Gen2 of the Raine study, our study addressed the following aims:

1. To determine if early childhood development in separate or multiple domains of development in the first 3 years of life is associated with HE in childhood and adolescence.
2. To compare these associations with those of early childhood development and anxiety/depression in childhood and adolescence.
3. To investigate whether the presence of early childhood developmental delays are associated with recurrence of HE in adolescence, which is a marker for poorer outcomes.

## Methods

### Participants

Ethical approval for the study was granted by the University of Western Australia Human Research Ethics Committee (HREC).

Data were taken from The Raine Study, a pregnancy cohort study that began in 1989, in which 2900 pregnant women were enrolled with an average recruitment age of 18 weeks of gestation, from the public antenatal clinic at the major obstetric hospital in Perth, Western Australia, and nearby private practices. The ongoing study has followed 2446 infants born over a 3-year period, with numerous follow-up assessments in childhood and adolescence. Inclusion criteria for mothers' participation in the study included gestational age between 16 and 20 weeks, proficiency in English and an intention to remain in Western Australia so to allow for long-term participation. Demographic and social information of parents/guardians was collected throughout the study. The rate of attrition in the Raine study is 29% from age 1 year to age 17 years (Straker et al., 2017). Participants are representative of the general Western Australian population at the time of recruitment and into infancy (Straker et al., 2017).

### Measures

#### Early childhood development

The Infant Monitoring Questionnaire (Bricker, Squires, Kaminski, & Mounts, 1988) (IMQ; now known as the Ages and Stages Questionnaire) was completed by parents/guardians at ages 1, 2 and 3 years. The IMQ is a screening tool for monitoring early childhood development in five broad domains: communication, gross motor, fine motor, adaptive (problem-solving) and personal-social. A 35-item version of the IMQ was administered at ages 1 and 2 years, and a 30-item version was administered at age 3 years. Upon agreement with the authors, age 1 and age 2 IMQ data were condensed to 30-items for standardisation within the analyses, due to the inclusion of one item that is 150 developmental quotient in each domain. This item was therefore removed from each domain at age 1 and age 2 years, and no significant differences were noted. Parents were asked to assess their child's behaviour by domain, and score ‘yes’ if the child does behave in such way, ‘sometimes’ if the child occasionally behaves in such way or ‘no’ if the child does not behave in such way. Higher scores indicate better development. The IMQ contain age-specific developmental items. The IMQ has good criterion validity, sensitivity, specificity and test–retest reliability (Bricker et al., 1988). The IMQ also has good criterion validity when compared with the Bayley Scales of Infant Development, and the Stanford–Binet Intelligence Test (*r* = 0.89) (Bricker & Squires, 1989) and is endorsed for use by the American Academy of Pediatrics (Schonhaut, Armijo, Schönstedt, Alvarez, & Cordero, 2013).

The IMQ was scored as per the instructions in the manual (Squires, 1993) and the suggested cut-off scores were used to define low-functioning infants and normal-functioning infants in each domain (see online Supplementary Table S3). Scoring below this cut-off was labelled as ‘Delay’, while scoring above this cut-off was labelled as ‘Average’. A further measure of low functioning was calculated based on scores within the lowest 10th percentile for each domain. The data were also dichotomised and labelled as ‘Low’ (⩽10 percentile) and ‘Average’ (⩾11 percentile).

#### Childhood/adolescent outcomes: hallucinatory experiences and anxiety/depression

The Child Behaviour Checklist (CBCL)/Youth Self-Report (YSR) (Achenbach, 1991; Achenbach & Edelbrock, 1983) are questionnaires used to measure emotional and behavioural problems in children and adolescents, and contain comparable items (Achenbach, Dumenci, & Rescorla, 2002). They include eight scales: anxious/depressed, depressed, somatic complaints, social problems, thought problems, attention problems, rule-breaking behaviour and aggressive behaviour. For the purpose of the current analyses, we use CBCL/YSR data collected at ages 10 years (parent-report) and 14 and 17 (self-report) years.

HE were indexed by two items on the ‘thought problems’ subscale of the CBCL/YSR, ‘I hear sounds or voices that other people think aren't there’ (item 40) and ‘I see things that other people think aren't there’ (item 70). These items have been found to have good predictive power for measuring PE (Kelleher et al., 2011). HE were considered as the endorsement of ‘somewhat/sometimes true’ or ‘very true/often true’ for either or both items, at one or more of the three time-points. Subgroups of those with transient experiences (reported at one time-point only) and recurrent experiences (reported at more than one time-point) were also created.

Anxious/depressed symptomology was determined using standardised *t*-scores from the CBCL/YSR anxious/depressed subscale. A cut-off point for *t*-scores over and including 70 was applied, deemed to be clinically significant by Achenbach (1991). Participants with a *t*-score of ⩾70 at one or more of the three time-points (ages 10, 14 and 17 years) and who had never reported PE were assigned to the anxious/depressed group for comparison.

#### Covariates

Covariates entered in the analyses included child's age (in months) at the time of their developmental assessment; maternal highest qualification, birthweight, sex at birth and family income at the time of the developmental assessments. Maternal highest qualification was measured when the participants were aged 8 years and was scaled from 0 to 8, and ranged from no qualification to postgraduate diploma/higher degree. Family income was measured at ages 1, 2 and 3 years and was based on five categories (less than $7000, $7000–11 999, $12 000–23 999, $24 000–35 999 and more than $36 000).

### Statistical analysis

Participants were divided into the HE group (those who reported PE at any time-point) and the anxious/depressed group (those who scored above the cut-off on the CBCL/YSR at any time-point). Controls were defined as those who did not report HE nor had anxious/depressed symptoms above the cut-off at any time-point. The HE group was subdivided into those with transient HE (those who reported HE at one time-point only) and recurrent HE (those who reported PE at either two or three time-points).

The HE and anxious/depressed groups were compared to control participants on demographic characteristics using independent *t* tests and a chi-squared test. Raw IMQ scores were converted to *z*-scores, in order to standardise each version administered at ages 1, 2 and 3 years. The data were analysed using complete case approaches.

Using Stata ver. 16 (StataCorp, 2009), we fitted a series of mixed-effect logistic models with a random effect accounting for the within-subject variance. We report the main effects of early childhood development in the first 3 years of life and later outcomes in childhood and/or adolescence. The exposure variables were the five subscales of the IMQ (communication, gross motor, fine motor, adaptive and personal-social) at ages 1, 2 and 3 years and the primary outcome variables were HE and anxious/depressed group membership. This approach allowed for the identification of associations between developmental scores and HE or anxious/depressed symptoms by detecting unique variances within the scores after accounting for within- and between-subject variance. Each of the aforementioned covariates was adjusted for throughout all of the analyses.

Developmental time-specific investigations were then conducted using separate logistic regression models (at ages 1, 2 and 3 years, respectively) to investigate the association between early childhood development at specific ages and later HE or anxious/depressed symptoms.

Cumulative risk based on multiple domain delays was also calculated. Cut-off scores for the IMQ were applied at each year (ages 1, 2 and 3 years). Three categories were created, 0 domain delays (reference group), 1 or 2 domain delays and 3–5 (multiple) domain delays, where the outcome variables were HE and anxious/depressed. Cumulative risk at age 3 years based on scores within the lowest 10th percentile on each domain was also analysed.

All analyses were also performed to determine associations between the early childhood developmental scores and subgroups of HE, transient and recurrent.

## Results

Of the 2446 participants who initially took part, data from *n* = 1101 participants were available for the present analysis. An attrition analysis is presented in online Supplementary Table S1. Those who were included in the present analysis were younger, had a higher birthweight and better overall development at age 2 years, as well as having mothers with higher qualifications, than those who were not included.

### Hallucinatory experiences

The prevalence of HE was 11.1% in the overall cohort. Among the participants in the HE group (*n* = 228), 48% reported at age 17; 38% reported at age 14 and 14% at age 10 (parent-report). At age 10 years, auditory HE were reported at higher rates (83%) while at 14 years, visual HE were more prevalent (84%). At age 17 years, rates of auditory and visual HE were comparable (50% respectively), and 48% of those who reported HE at this age reported both. Transient experiences were reported in 82% of cases, while recurrent experiences were reported in 18% of cases. A total of *n* = 40 participants reported HE at two time-points, and a further *n* = 2 participants reported HE at three time-points.

### Anxious/depressed

The prevalence of anxious/depressed symptoms at or above cut-off was 5.6% in the overall cohort. A total of *n* *=* 114 participants scored on or above cut-off at least once at ages 10, 14 or 17 years. Seven (6%) had symptoms at more than one time-point. A total of 32% had symptoms at age 17; 18% had symptoms at age 14 and 32% had symptoms at age 10. Of note, *n* *=* 43 (37.7%) of the anxious/depressed group also reported PE, and were therefore only included in the HE group.

No significant group differences in demographic characteristics were found between the HE and control groups, with the exception of maternal highest level of education (*t* = −3.80, *p* < 0.01) and family income at age 1 (*t* = −3.76, *p* < 0.01), where less maternal education and a lower family income at age 1 were noted for the HE group. No significant group differences were found between the anxious/depressed and control groups (see Table 1).

**Table 1.** Demographic characteristics for the HE group and anxious/depressed groups when compared to the control group

	Descriptive statistics		Analysis (reference: control group)				
	HE group Mean (s.d.)	Anxious/depressed group Mean (s.d.)	Control group Mean (s.d.)	HE group χ^2^ or *t*	*p-value*	Anxious/depressed group χ^2^ or *t*	*p-value*
Demographic variable							
Sex at birth, *n* (%)				1.03	0.31	1.66	0.20
Males	119 (52%)	40 (56%)	388 (48%)				
Females	109 (49%)	31 (44%)	414 (52%)				
Ethnicity^a^							
Caucasian (European descent)	203 (91%)	69 (99%)	703 (90%)				
Aboriginal	1	0	5				
Polynesian	0	0	8				
Vietnamese	0	0	4				
Chinese	13	0	37				
Indian	6	1	20				
Other	0	0	7				
Birthweight (in g)	3281.7 (671.8)	3281.7 (626.2)	3341.3 (560.1)	−1.35	0.18	−0.09	0.93
Age 1 (months)	1.50 (0.11)	1.14 (0.11)	1.14 (0.10)	−0.70	0.48	0.21	0.84
Age 2	2.13 (0.15)	2.16 (0.18)	2.13 (0.14)	0.07	0.94	−1.46	0.14
Age 3	3.15 (0.16)	3.16 (0.18)	3.12 (0.12)	−3.13	**0.002**	−2.94	**0.003**
Age 10	10.59 (0.20)	10.62 (0.17)	10.57 (0.17)	−1.39	0.17	−2.14	**0.03**
Age 14	13.99 (0.21)	14.02 (0.23)	13.99 (0.19)	−0.70	0.48	−1.26	0.21
Age 17	17.07 (0.27)	17.03 (0.29)	17.02 (0.22)	−2.80	**0.005**	−0.39	0.70
Maternal highest qualification	2.42 (2.49)	2.60 (2.35)	3.14 (2.30)	−3.80	**<0.001**	−1.84	0.07
Family income age 1	2.77 (1.20)	2.78 (1.40)	3.11 (1.24)	−3.76	**<0.001**	−1.90	0.06
Family income age 2	2.83 (1.70)	3.03 (2.11)	3.05 (1.41)	−1.61	0.11	−0.07	0.94
Family income age 3	2.95 (1.63)	2.80 (1.61)	3.10 (1.32)	−1.23	0.22	−1.74	0.08

s.d., standard deviation.

a Some missing data.

### Association between early childhood development and PE

Mean scores for the IMQ subscales at each age (1, 2 and 3 years) for the HE, anxious/depressed and control groups, including the subgroups of the HE group (transient and recurrent HE), are presented in online Supplementary Table S2.

In the overall random effects model, which accounted for development over the first 3 years of age, there was no significant association between any of developmental domains and HE. In the age-specific analysis, lower adaptive scores at age 1 year were associated with HE. At age 2 years, lower scores in 4 of the 5 developmental domains (with the exception of gross motor) were associated with HE. At age 3 years, lower scores in any of the five domains (communication, gross motor, fine motor, adaptive and personal-social) were associated with HE (see Table 2).

**Table 2.** Overall and age-specific developmental scores between ages 1 and 3 and risk of later HE (*n* = 228) [also subdivided into transient (*n* = 114) or recurrent (*n* = 42)] and anxiety/depression (*n* = 71) (compared with Controls (*n* = 802) (reference group is controls)

	Communication	Gross motor	Fine motor	Adaptive	Personal-social
	OR (CI)	OR (CI)	OR (CI)	OR (CI)	OR (CI)
HE group					
Mixed effect model	1.04 (0.92–1.19)	1.02 (0.90–1.07)	0.99 (0.84–1.16)	1.05 (0.92–1.05)	1.01 (0.87–1.18)
Age 1 year	1.14 (0.96–1.35)	1.09 (0.93–1.27)	1.08 (0.93–1.28)	**1.27** (1.08–1.49)	1.10 (0.94–1.30)
Age 2 years	**1.39** (1.18–1.64)	1.16 (0.99–1.39)	**1.23** (1.03–1.47)	**1.39** (1.18–1.66)	**1.20** (1.01–1.45)
Age 3 years	**1.32** (1.12–1.54)	**1.25** (1.06–1.47)	**1.32** (1.14–1.54)	**1.35** (1.16–1.56)	**1.20** (1.02–1.43)
Anxious/depressed group					
Mixed effect model	0.98 (0.63–1.54)	1.19 (0.78–1.79)	1.12 (0.72–1.75)	1.17 (0.76–1.82)	1.19 (0.76–1.85)
Age 1 year	1.02 (0.77–1.35)	0.91 (0.68–1.22)	1.01 (0.76–1.33)	1.01 (0.76–1.35)	1.20 (0.93–1.56)
Age 2 years	0.92 (0.65–1.28)	1.20 (0.93–1.54)	1.20 (0.92–1.58)	1.30 (1.00–1.72)	1.08 (0.79–1.47)
Age 3 years	1.05 (0.79–1.39)	**1.39** (1.11–1.75)	1.20 (0.93–1.54)	1.14 (0.88–1.47)	1.19 (0.90–1.56)
Transient HE group					
Mixed effect model	1.03 (0.60–1.78)	1.05 (0.63–1.75)	1.02 (0.62–1.66)	1.28 (0.85–1.92)	1.08 (0.72–1.59)
Age 1 year	1.05 (0.84–1.32)	0.94 (0.76–1.19)	0.89 (0.70–1.12)	**1.33** (1.08–1.64)	1.00 (0.80–1.23)
Age 2 years	1.12 (0.88–1.43)	1.06 (0.85–1.33)	0.97 (0.76–1.25)	**1.28** (1.02–1.61)	1.05 (0.82–1.35)
Age 3 years	1.14 (0.93–1.41)	1.16 (0.93–1.45)	**1.33** (1.00–1.61)	**1.27** (1.04–1.54)	1.15 (0.92–1.43)
Recurrent HE group					
Mixed effect model	**2.04** (1.14–3.70)	1.72 (0.79–3.85)	1.72 (0.96–3.03)	1.37 (0.78–2.43)	1.47 (0.81–2.70)
Age 1 year	1.39 (0.99–1.92)	**1.37** (1.06–1.75)	**1.43** (1.09–1.89)	1.19 (0.86–1.63)	1.30 (0.95–1.78)
Age 2 years	**2.17** (1.54–3.03)	1.28 (0.90–1.82)	**2.04** (1.43–2.94)	**1.44** (1.01–2.08)	**1.56** (1.06–2.27)
Age 3 years	**1.85** (1.45–2.38)	**1.64** (1.27–2.17)	1.82 (0.98–1.82)	**1.41** (1.06–1.92)	**1.59** (1.14–2.17)

OR, odds ratio; CI, confidence intervals.

Bolded text represents analyses where 95% CI do not cross 1.

Odds ratios are adjusted for sex at birth, age, birthweight, maternal highest qualification and family income at each year.

These results are presented, stratified by gender, in Tables S4 and S5 of the online Supplementary materials.

### Association between early childhood development and anxious/depressed symptoms

No significant associations between any of developmental domains and anxious/depressed symptoms were found. In the age-specific analysis, only lower gross motor scores at age 3 years were associated with anxious/depressed symptoms (see Table 2).

### Association between early childhood development and transient and recurrent HE

No significant associations between any of developmental domains and transient HE were found. In the age-specific analysis, lower adaptive scores at ages 1, 2 and 3 years, as well as lower fine motor scores at age 3 years, were significantly associated with transient HE.

**Fig. 1.**
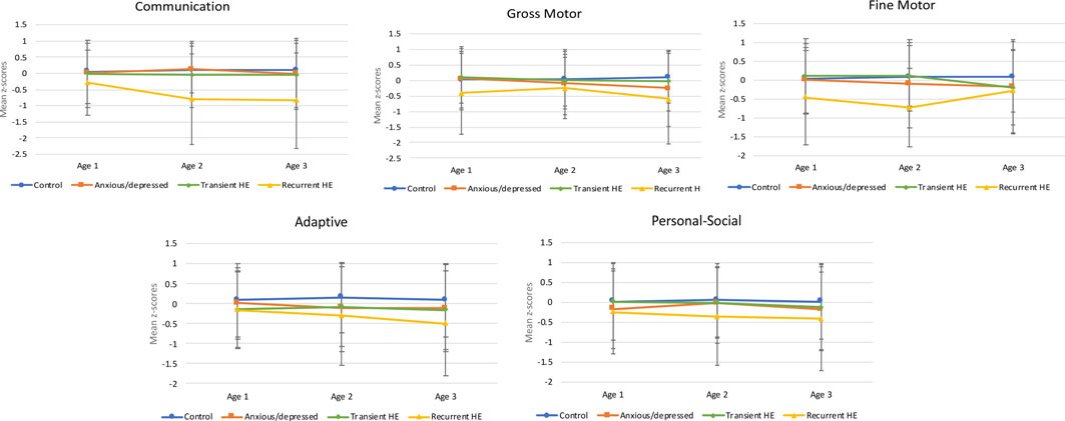
Longitudinal unadjusted mean *z*-scores with standard deviation bars for each developmental domain (ages 1, 2 and 3 years) for all groups.

Lower communication scores in the first 3 years were associated with recurrent HE. In the age-specific analysis, lower gross motor and fine motor scores at age 1 year were significantly associated with recurrent HE. At age 2 years, lower scores in 4 of the 5 developmental domains (with the exception of gross motor) were associated with recurrent HE. At age 3 years, lower scores in 4 of the 5 early childhood development domains (communication, gross motor, adaptive and personal-social) were significantly associated with recurrent HE. All data are presented in Fig. 1.

### Cumulative risk based on multiple domain delays

Based on cut-off scores, having 3–5 developmental domain delays at age 2 led to an almost 5-fold increased risk for HE (adjusted odds ratio 4.86, 95% confidence interval 1.69–14.05, *p* = 0.004). Having 1–2 developmental domain delays at age 3 led to an almost 2-fold increased risk for HE and having multiple domain delays increased the risk of HE over 3-fold (see Table 3). Among young people who reported recurrent HE, 30.9% had developmental domain delays at age 2 years and 40.5% had developmental domain delays at age 3 years.

**Table 3.** Cumulative risk for later HE (*n* = 228) [Transient (*n* = 114) or recurrent (*n* = 42)] and anxiety/depression (*n* = 71) based on number of developmental delays (below cut-off) at ages 1, 2 and 3 years for each group

Cumulative risk (0 delays reference category)	1–2 delays *N* (% below cut-off)	OR (CI)	*p*-value	3–5 delays *N* (% below cut-off)	OR (CI)	*p*-value
Age 1						
HE group	41 (18)	**1.70** (1.10–2.62)	**0.02**	4 (1.8)	0.75 (0.16–3.52)	0.72
Anxious/depressed group	8 (11.3)	0.85 (0.37–1.95)	0.70	0	–	–
Transient HE group^a^	17 (14.9)	1.46 (0.82–2.61)	0.20	1 (0.9)	0.75 (0.09–5.99)	0.78
Recurrent HE group^a^	9 (21.4)	1.88 (0.83–4.23)	0.13	0	–	–
Controls	86 (10.7)			10 (1.2)		
Age 2						
HE group	50 (21.9)	**1.81** (1.20–2.72)	**0.004**	8 (3.5)	**4.86** (1.69–14.05)	**0.004**
Anxious/depressed group	10 (14.1)	1.00 (0.49–2.08)	0.99	1 (1.4)	1.66 (0.19–14.08)	0.64
Transient HE group^a^	20 (17.5)	1.24 (0.72–2.13)	0.45	1 (0.9)	0.95 (0.11–8.02)	0.96
Recurrent HE group^a^	10 (23.8)	**3.49** (1.46–8.33)	**0.005**	3 (7.1)	**21.11** (4.22–105.91)	**<0.001**
Controls	118 (14.7)			7 (0.9)		
Age 3						
HE group	60 (26.3)	**1.80** (1.24–2.62)	**0.002**	17 (7.5)	**3.60** (1.70–7.23)	**0.001**
Anxious/depressed group	18 (25.4)	1.53 (0.84–2.78)	0.17	3 (4.2)	1.31 (0.29–5.97)	0.72
Transient HE group^a^	38 (33.3)	**2.17** (1.38–3.42)	**0.001**	3 (2.6)	1.33 (0.37–4.70)	0.66
Recurrent HE group^a^	10 (23.8)	1.45 (0.64–3.31)	0.37	7 (16.7)	**7.68** (2.73–21.58)	**<0.001**
Controls	154 (19.2)			19 (2.4)		

a Subgroups of the HE group.

In the percentile analysis, delays (lowest 10th percenile for all participants) in 1–2 domains led to over a 2-fold increase risk for HE, while delays in multiple domains led to over a 3-fold increased risk for HE (see Table 4). For the HE subgroups, delays (by percentile) in 1–2 developmental domains led to over a 2-fold increased risk for transient HE. Delays in 3–5 developmental domains led to over a 7-fold increased risk for recurrent HE.

**Table 4.** Cumulative risk for later HE (transient or recurrent) and anxious/depressed based on number of domain delays at age 3 years for each group

Cumulative risk (0 delays reference category)	1–2 domains OR (CI)	*p*-value	3–5 domains OR (CI)	*p*-value
HE group	2.21 (1.54–3.18)	**<0.001**	3.60 (1.82–6.99)	**<0.001**
Anxious/depressed group	1.57 (0.87–2.81)	0.13	1.07 (0.24–4.78)	0.93
Transient HE group^a^	2.31 (1.48–3.60)	**<0.001**	1.54 (0.51–4.67)	0.16
Recurrent HE group^a^	1.98 (0.92–4.25)	0.08	7.23 (2.62–19.98)	**<0.001**

OR, odds ratio; CI, confidence intervals.

Statistically significant results (*p* < 0.05) are presented in bold text.

a Subgroups of the HE group.

Multiple developmental domain delays did not lead to a significant increase in cumulative risk for anxious/depressed symptoms, in either the cut-off or percentile analysis.

## Discussion

In this study, we examined longitudinally the association between early childhood development and HE in childhood and adolescence in a population-based, representative sample of young people. Participants who reported HE in childhood and/or adolescence showed lower early developmental scores from ages 1 and 2 years on a range of measures. By age 3 years, lower scores in any of the five developmental domains were significantly associated with the risk of later reporting HE. Cumulative risk based on multiple (3–5) domain delays at age 3 years led to over a 3-fold increased risk for HE. For those participants who would later report clinically significant anxious/depressed symptomology, no association was found for overall development over the first 3 years, with the exception of gross motor scores at age 3, and there was also no increased cumulative risk based on multiple domains delays.

Further analyses within the HE group showed that when subdividing the group by transient and recurrent experiences, lower adaptive scores at ages 1, 2 and 3 years were specifically associated with transient HE. Delays in 1 or 2 developmental domains at age 3 years led to over a 2-fold increased risk for transient HE. Stronger effects were found for those who reported recurrent HE, in that a greater number of associations were found with early developmental scores compared to those who only reported HE at one time-point in childhood or adolescence. Both at ages 2 and 3 years, lower scores in 4 of the 5 developmental domains were associated with recurrent HE. At age 3 years, over 40% of the recurrent HE group had at least one developmental delay, as measured by cut-off scores. Multiple (3–5) domain delays led to over a 7-fold increased risk. These findings convey the strong associations between poorer early childhood development and mental health outcomes later in life. Our findings indicate a specific risk associated with the psychosis spectrum, and a strong association with recurrent HE throughout childhood and adolescent development.

The present study has extended previous research on early childhood development and PE. Using the Denver Developmental Screening Test (which includes four measures of early development: fine and gross motor, social and communication skills), Hameed et al. (2018) showed that declining communication and social skills in the first 4 years of life were significantly associated with PE at one time-point at age 12 years. Our study has extended these findings by measuring adaptive (problem-solving) skills, and by examining HE over three time-points in childhood and adolescence. In addition, we explored the comparison between the associations between poorer early development and later HE and clinically significant anxious/depressed symptomology as outcomes. Lower scores in gross motor development at age 3 years were associated with later clinically significant anxious/depressed symptomology. Although the co-occurrence of PE and anxiety/depression has been established (Wigman et al., 2011), our findings suggest that poorer development is driven by variability associated with psychosis spectrum symptomology rather than psychopathology more generally, a pattern that has also been shown in other studies (Cannon et al., 2002).

Our findings pertaining to early infant communication development and its relationship with HE, in particular recurrent HE, are in-line with those of Hameed et al. (2018). Impairments in language abilities and the dysconnectivity of language networks have been a central topic in the research of cognitive dysfunction in psychosis, and are thought to contribute to the development of auditory hallucinations (Benetti et al., 2015). Altered white matter in language areas has also been seen in young people with PE (Dooley et al., 2020). Our results suggest that language and communication deficits may be detectable as early as the first year of life in young people at risk for psychosis.

Poorer early adaptive development or problem-solving skills were also associated with HE, and in particular, transient HE. The transient HE group were characterised by persistently lower scores in adaptive skills throughout the first 3 years. Reasoning, control and planning are core to adaptive development and delays in these areas have been described in the later stages of childhood in individuals who go on to develop schizophrenia (Reichenberg et al., 2009). Transient HE, or once-off PE, are linked to poorer global functioning throughout adolescence and early adulthood (Healy et al., 2018).

The recurrent HE group had poorer developmental scores in both cognitive and motor domains throughout the first 3 years of life. Motor dysfunction may be reflective of a core feature of psychosis and schizophrenia. Psychomotor developmental deviations are more marked in those later diagnosed with schizophrenia, when compared those later diagnosed with bipolar disorder, and have an earlier onset (Parellada, Gomez-Vallejo, Burdeus, & Arango, 2017). Keskinen et al. (2015) hypothesised that delayed motor development may be a marker of other risk processes that interact with genetic liability for psychosis and schizophrenia. Murray et al. (2006) retrospectively found that individuals with schizophrenia had early delays in neuromotor milestones. Motor development therefore may be an important trait marker for recurrent HE, which is of important clinical utility, as young people with persistent symptoms have poorer functional outcomes (Healy et al., 2018), higher levels of stress reactivity (Collip et al., 2013) and are at greater risk of conversion to psychosis (Dominguez, Wichers, Lieb, Wittchen, & van Os, 2011). Recurrence is thought to be indicative of a more serious underlying psychopathological process, and represents an important, early, non-specific marker of later mental health problems (Kelleher et al., 2012b).

Strengths of the study include the availability of very early developmental data from infancy from a population-based longitudinal birth cohort and the use of a robust and widely-used assessment of early childhood development, as well as the consistency of measures used across time-points. The HE group also included those with clinically significant anxious/depressed symptomology, due to the established co-occurrence (Kounali et al., 2014) and known overlap with psychopathology more generally (Trotta et al., 2020). Kounali and colleagues also found that markers of abnormal neurodevelopment showed stronger association with PE, when compared to depression. However, limitations of the study should also be noted. The measurement of HE was confined to the self-report of two types of symptomology (namely, auditory and visual HE); however, these are the most common and validated in childhood community samples (Laurens, Hobbs, Sunderland, Green, & Mould, 2012). HE were indexed by responses ‘somewhat/sometimes true’ or ‘very true/often true’ which may have been inflated prevalence rates. Furthermore, at age 10 years, a parent-report of HE was used which may have led to an underestimation of HE. The prevalence of clinically significant anxious/depressed symptoms might also be underestimated, particularly as a high proportion who also reported HE were counted within the HE group rather than the Anxious/depressed group as this was the main focus of our analysis. Within the analyses, the omission of parental psychopathology as a covariate must also be noted. This was not consistently measured throughout The Raine Study. Furthermore, the impact of multiple comparisons should be considered, particularly for the results in the anxious/depressed group. Finally, attrition rates within The Raine Study may have led to follow-up bias; however, this may have led to underestimation rather than over estimation of effect sizes since those who were lost to follow-up had poorer development at age 2 years.

In conclusion, our study has shown that cognitive and motor developmental delays can be detected from infancy in young people who go on to report HE in childhood and adolescence. Our findings are reminiscent of the ‘early insult’ theory in psychosis, where early genetic or environmental insults lead to aberrant brain development (Murray & Lewis, 1987) which is exacerbated thereafter by childhood trauma and adversity (Croft et al., 2019). Exploration of early childhood development as both a meaningful marker for later mental health outcomes and a viable critical period for early intervention is an exciting emerging area in psychosis research.

## Supplementary material

For supplementary material accompanying this paper visit https://doi.org/10.1017/S0033291721003883. click here to view supplementary material

## References

[ref1] Achenbach, T.M. (1991). Manual for the youth self-report and 1991 profile (pp. 1–13). Burlington: Department of Psychiatry, University of Vermont.

[ref2] Achenbach, T. M., Dumenci, L., & Rescorla, L. A. (2002). Ten-year comparisons of problems and competencies for national samples of youth: Self, parent, and teacher reports. Journal of Emotional and Behavioral Disorders, 10(4), 194–203.

[ref3] Achenbach, T. M., & Edelbrock, C. S. (1983). Manual for the child behavior checklist and revised child behavior profile.

[ref4] Benetti, S., Pettersson-Yeo, W., Allen, P., Catani, M., Williams, S., Barsaglini, A., … Mechelli, A. (2015). Auditory verbal hallucinations and brain dysconnectivity in the perisylvian language network: A multimodal investigation. Schizophrenia Bulletin, 41(1), 192–200. 24361862 10.1093/schbul/sbt172 PMC4266279

[ref5] Bricker, D., & Squires, J. (1989). The effectiveness of parental screening of at-risk infants: The infant monitoring questionnaires. Topics in Early Childhood Special Education, 9(3), 67–85.

[ref6] Bricker, D, Squires, J, Kaminski, R, & Mounts, L. (1988). The validity, reliability, and cost of a parent-completed questionnaire system to evaluate at-risk infants. Journal of pediatric psychology, 13(1), 55–68. 2455033 10.1093/jpepsy/13.1.55

[ref7] Burton, B. K., Hjorthøj, C., Jepsen, J. R., Thorup, A., Nordentoft, M., & Plessen, K. J. (2016). Research review: Do motor deficits during development represent an endophenotype for schizophrenia? A meta-analysis. Journal of Child Psychology and Psychiatry, 57(4), 446–456. 26577292 10.1111/jcpp.12479

[ref8] Calkins, M. E., Moore, T. M., Satterthwaite, T. D., Wolf, D. H., Turetsky, B. I., Roalf, D. R., … Gur, R. C. (2017). Persistence of psychosis spectrum symptoms in the Philadelphia neurodevelopmental cohort: A prospective two-year follow-up. World Psychiatry, 16(1), 62–76. 28127907 10.1002/wps.20386 PMC5269480

[ref9] Cannon, M., Caspi, A., Moffitt, T. E., Harrington, H., Taylor, A., Murray, R. M., & Poulton, R. (2002). Evidence for early-childhood, pan-developmental impairment specific to schizophreniform disorder: Results from a longitudinal birth cohort. Archives of General Psychiatry, 59(5), 449–456. 11982449 10.1001/archpsyc.59.5.449

[ref10] Carey, E., Dooley, N., Gillan, D., Healy, C., Coughlan, H., Clarke, M., … Cannon, M. (2019). Fine motor skill and processing speed deficits in young people with psychotic experiences: A longitudinal study. Schizophrenia Research, 204, 127–132. 30174253 10.1016/j.schres.2018.08.014

[ref11] Carey, E., Gillan, D., Healy, C., Dooley, N., Campbell, D., McGrane, J., … Cannon, M. (2021). Early adult mental health, functional and neuropsychological outcomes of young people who have reported psychotic experiences: A 10-year longitudinal study. Psychological Medicine, 51(11), 1861–1869. 32216843 10.1017/S0033291720000616

[ref12] Carter, A. S., Briggs-Gowan, M. J., & Davis, N. O. (2004). Assessment of young children's social-emotional development and psychopathology: Recent advances and recommendations for practice. Journal of Child Psychology and Psychiatry, 45(1), 109–134. 14959805 10.1046/j.0021-9630.2003.00316.x

[ref13] Caspi, A., Houts, R. M., Ambler, A., Danese, A., Elliott, M. L., Hariri, A., … Ramrakha, S. (2020). Longitudinal assessment of mental health disorders and comorbidities across 4 decades among participants in the Dunedin birth cohort study. JAMA Network Open, 3(4), e203221–e203221. 32315069 10.1001/jamanetworkopen.2020.3221 PMC7175086

[ref14] Cederlöf, M., Kuja-Halkola, R., Larsson, H., Sjölander, A., Östberg, P., Lundström, S., … Lichtenstein, P. (2017). A longitudinal study of adolescent psychotic experiences and later development of substance use disorder and suicidal behavior. Schizophrenia Research, 181, 13–16. 27615409 10.1016/j.schres.2016.08.029

[ref15] Clarke, M. C., Tanskanen, A., Huttunen, M., Leon, D. A., Murray, R. M., Jones, P. B., & Cannon, M. (2011). Increased risk of schizophrenia from additive interaction between infant motor developmental delay and obstetric complications: Evidence from a population-based longitudinal study. American Journal of Psychiatry, 168(12), 1295–1302. 21890789 10.1176/appi.ajp.2011.11010011

[ref16] Collip, D., Wigman, J. T., Myin-Germeys, I., Jacobs, N., Derom, C., Thiery, E., … van Os, J. (2013). From epidemiology to daily life: Linking daily life stress reactivity to persistence of psychotic experiences in a longitudinal general population study. PLoS ONE, 8(4), e62688. 23626848 10.1371/journal.pone.0062688 PMC3633877

[ref17] Croft, J., Heron, J., Teufel, C., Cannon, M., Wolke, D., Thompson, A., … Zammit, S. (2019). Association of trauma type, age of exposure, and frequency in childhood and adolescence with psychotic experiences in early adulthood. JAMA Psychiatry, 76(1), 79–86. 30477014 10.1001/jamapsychiatry.2018.3155 PMC6490231

[ref18] Dominguez, M., Wichers, M., Lieb, R., Wittchen, H.-U., & van Os, J. (2011). Evidence that onset of clinical psychosis is an outcome of progressively more persistent subclinical psychotic experiences: An 8-year cohort study. Schizophrenia Bulletin, 37(1), 84–93. 19460881 10.1093/schbul/sbp022 PMC3004179

[ref19] Dooley, N., O'Hanlon, E., Healy, C., Adair, A., McCandless, C., Coppinger, D., … Frodl, T. (2020). Psychotic experiences in childhood are associated with increased structural integrity of the left arcuate fasciculus – A population-based case-control study. Schizophrenia Research, 215, 378–384. 31495700 10.1016/j.schres.2019.08.022

[ref20] Green, M. J., Tzoumakis, S., Laurens, K. R., Dean, K., Kariuki, M., Harris, F., … Carr, V. J. (2019). Early developmental risk for subsequent childhood mental disorders in an Australian population cohort. Australian & New Zealand Journal of Psychiatry, 53(4), 304–315. 30501395 10.1177/0004867418814943

[ref21] Gur, R. C., Calkins, M. E., Satterthwaite, T. D., Ruparel, K., Bilker, W. B., Moore, T. M., … Gur, R. E. (2014). Neurocognitive growth charting in psychosis spectrum youths. JAMA Psychiatry, 71(4), 366–374. 24499990 10.1001/jamapsychiatry.2013.4190

[ref22] Hameed, M. A., Lingam, R., Zammit, S., Salvi, G., Sullivan, S., & Lewis, A. J. (2018). Trajectories of early childhood developmental skills and early adolescent psychotic experiences: Findings from the ALSPAC UK birth cohort. Frontiers in Psychology, 8, 2314. 29375433 10.3389/fpsyg.2017.02314 PMC5767306

[ref23] Healy, C., Brannigan, R., Dooley, N., Coughlan, H., Clarke, M., Kelleher, I., … Cannon, M. (2019). Childhood and adolescent psychotic experiences and risk of mental disorder: A systematic review and meta-analysis. Psychological Medicine, 49(10), 1589–1599. 31088578 10.1017/S0033291719000485

[ref24] Healy, C., Campbell, D., Coughlan, H., Clarke, M., Kelleher, I., & Cannon, M. (2018). Childhood psychotic experiences are associated with poorer global functioning throughout adolescence and into early adulthood. Acta Psychiatrica Scandinavica, 138(1), 26–34. 29855047 10.1111/acps.12907

[ref25] Honings, S., Drukker, M., Groen, R., & van Os, J. (2016). Psychotic experiences and risk of self-injurious behaviour in the general population: A systematic review and meta-analysis. Psychological Medicine, 46(2), 237–251. 26419206 10.1017/S0033291715001841

[ref26] Irwin, L. G., Siddiqi, A., & Hertzman, G. (2007). Early child development: A powerful equalizer. Human Early Learning Partnership (HELP) Vancouver, BC.

[ref27] Kelleher, I., Cederlöf, M., & Lichtenstein, P. (2014). Psychotic experiences as a predictor of the natural course of suicidal ideation: A Swedish cohort study. World Psychiatry, 13(2), 184–188. 24890071 10.1002/wps.20131 PMC4102291

[ref28] Kelleher, I., Connor, D., Clarke, M. C., Devlin, N., Harley, M., & Cannon, M. (2012a). Prevalence of psychotic symptoms in childhood and adolescence: A systematic review and meta-analysis of population-based studies. Psychological Medicine, 42(9), 1857–1863. 22225730 10.1017/S0033291711002960

[ref29] Kelleher, I., Harley, M., Murtagh, A., & Cannon, M. (2011). Are screening instruments valid for psychotic-like experiences? A validation study of screening questions for psychotic-like experiences using in-depth clinical interview. Schizophrenia Bulletin, 37(2), 362–369. 19542527 10.1093/schbul/sbp057 PMC3044617

[ref30] Kelleher, I., Keeley, H., Corcoran, P., Lynch, F., Fitzpatrick, C., Devlin, N., … Harley, M. (2012b). Clinicopathological significance of psychotic experiences in non-psychotic young people: Evidence from four population-based studies. The British Journal of Psychiatry, 201(1), 26–32. 22500011 10.1192/bjp.bp.111.101543

[ref31] Keskinen, E., Marttila, A., Marttila, R., Jones, P., Murray, G., Moilanen, K., … Jääskeläinen, E. (2015). Interaction between parental psychosis and early motor development and the risk of schizophrenia in a general population birth cohort. European Psychiatry, 30(6), 719–727. 26070841 10.1016/j.eurpsy.2015.04.006 PMC4623356

[ref32] Kounali, D., Zammit, S., Wiles, N., Sullivan, S., Cannon, M., Stochl, J., … Heron, J. (2014). Common versus psychopathology-specific risk factors for psychotic experiences and depression during adolescence. Psychological Medicine, 44(12), 2557–2566. 25055173 10.1017/S0033291714000026 PMC4108252

[ref33] Laurens, K. R., Hobbs, M., Sunderland, M., Green, M. J., & Mould, G. (2012). Psychotic-like experiences in a community sample of 8000 children aged 9 to 11 years: An item response theory analysis. Psychological Medicine, 42(7), 1495–1506. 21999924 10.1017/S0033291711002108

[ref34] Maijer, K., Begemann, M. J., Palmen, S. J., Leucht, S., & Sommer, I. E. (2018). Auditory hallucinations across the lifespan: A systematic review and meta-analysis. Psychological Medicine, 48(6), 879–888. 28956518 10.1017/S0033291717002367

[ref35] Merikangas, K. R., He, J.-P., Burstein, M., Swanson, S. A., Avenevoli, S., Cui, L., … Swendsen, J. (2010). Lifetime prevalence of mental disorders in US adolescents: Results from the national comorbidity survey replication–adolescent supplement (NCS-A). Journal of the American Academy of Child & Adolescent Psychiatry, 49(10), 980–989. 20855043 10.1016/j.jaac.2010.05.017 PMC2946114

[ref36] Mollon, J., David, A. S., Morgan, C., Frissa, S., Glahn, D., Pilecka, I., … Reichenberg, A. (2016). Psychotic experiences and neuropsychological functioning in a population-based sample. JAMA Psychiatry, 73(2), 129–138. 26720073 10.1001/jamapsychiatry.2015.2551

[ref37] Murray, G., Jones, P., Moilanen, K., Veijola, J., Miettunen, J., Cannon, T., & Isohanni, M. (2006). Infant motor development and adult cognitive functions in schizophrenia. Schizophrenia Research, 81(1), 65–74. 16300931 10.1016/j.schres.2005.08.016

[ref38] Murray, R. M., & Lewis, S. W. (1987). Is schizophrenia a neurodevelopmental disorder? British Medical Journal (Clinical Research Ed.), 295(6600), 681. 3117295 10.1136/bmj.295.6600.681 PMC1247717

[ref39] Parellada, M., Gomez-Vallejo, S., Burdeus, M., & Arango, C. (2017). Developmental differences between schizophrenia and bipolar disorder. Schizophrenia Bulletin, 43(6), 1176–1189. 29045744 10.1093/schbul/sbx126 PMC5737496

[ref40] Reichenberg, A., Caspi, A., Harrington, H., Houts, R., Keefe, R. S., Murray, R. M., … Moffitt, T. E. (2009). Static and dynamic cognitive deficits in childhood preceding adult schizophrenia: A 30-year study. American Journal of Psychiatry, 167(2), 160–169. 10.1176/appi.ajp.2009.09040574 PMC3552325 20048021

[ref41] Schonhaut, L., Armijo, I., Schönstedt, M., Alvarez, J., & Cordero, M. (2013). Validity of the ages and stages questionnaires in term and preterm infants. Pediatrics, 131(5), e1468–e1474. 23629619 10.1542/peds.2012-3313

[ref42] Squires, J. (1993). Infant/child monitoring questionnaires procedures manual. Oregon: University of Oregon Center of Human Development.

[ref43] Straker, L., Mountain, J., Jacques, A., White, S., Smith, A., Landau, L., … Eastwood, P. (2017). Cohort profile: The western Australian pregnancy cohort (Raine) study–generation 2. International Journal of Epidemiology, 46(5), 1384–1385j. 28064197 10.1093/ije/dyw308 PMC5837608

[ref44] Trotta, A., Arseneault, L., Caspi, A., Moffitt, T. E., Danese, A., Pariante, C., … Fisher, H. L. (2020). Mental health and functional outcomes in young adulthood of children with psychotic symptoms: A longitudinal cohort study. Schizophrenia Bulletin, 46(2), 261–271. 31361314 10.1093/schbul/sbz069 PMC7442396

[ref45] Wigman, J., Lin, A., Vollebergh, W. A., van Os, J., Raaijmakers, Q. A., Nelson, B., … Yung, A. (2011). Subclinical psychosis and depression: Co-occurring phenomena that do not predict each other over time. Schizophrenia Research, 130(1–3), 277–281. 21458235 10.1016/j.schres.2011.03.003

