# Supplementary material for: Evidence that infant and early childhood developmental impairments are associated with hallucinatory experiences: results from a large, population-based cohort study
Source: Psychol Med. 2021 Sep 29;53(5):2116–24. doi: 10.1017/S0033291721003883 (PMC10106299; doi:10.1017/S0033291721003883)
Supplement: Supplementary file 1 [file S0033291721003883sup001.docx]

Table 1. Attrition analysis of the demographic characteristics and total IMQ scores for those included (*n=*1,101) and not included (*n=*1,345) in the overall analysis

|  |  | Descriptive Statistics | | | Analysis | | |
| --- | --- | --- | --- | --- | --- | --- | --- |
|  | | | *Included*  ***Mean* (SD)** | *Missing*  ***Mean* (SD)** | | **χ^2^ or t** | ***p-value*** |
| **Demographic Variable** | | |  |  | |  |  |
| Sex at birth, n (%) | | |  |  | | 0.74 | .39 |
| Males | | | 547 (49.7%) | 677 (50.3%) | |  |  |
| Females | | | 554 (50.3%) | 668 (49.7%) | |  |  |
| Birthweight (in grams) | | | 3328.5 (589.1) | 3258.5 (639.9) | | 2.93 | **.003** |
| Age 1 (months) | | | 1.15 (.10) | 1.17 (.14) | | -5.31 | **<.001** |
| Age 2 | | | 2.14 (.14) | 2.15 (.15) | | -2.38 | **.02** |
| Age 3 | | | 3.12 (.14) | 3.14 (.16) | | -3.84 | **<.001** |
| Age 10 | | | 10.58 (.18) | 10.63 (.22) | | -5.15 | **<.001** |
| Age 14 | | | 13.99 (.20) | 14 (.22) | | -1.63 | .10 |
| Age 17 | | | 17.02 (.23) | 17.14 (.31) | | -7.87 | **<.001** |
| Maternal highest qualification | | | 2.96 (2.36) | 2.74 (2.58) | | 2.08 | **.04** |
| Family income age 1 | | | 3.02 (1.25) | 3.01 (1.61) | | 0.23 | .82 |
| Family income age 2 | | | 3.01 (1.52) | 2.91 (1.75) | | 1.41 | .16 |
| Family income age 3 | | | 3.05 (1.41) | 3.01 (1.83) | | 0.59 | .55 |
| Total IMQ age 1 | | | 244.6 (40.5) | 242.2 (45.2) | | 1.37 | .18 |
| Total IMQ age 2 | | | 258.9 (29.1) | 255.4 (33.8) | | 2.44 | **.01** |
| Total IMQ age 3 | | | 255.8 (39.0) | 254.3 (41.6) | | 0.84 | .38 |

*Note:* SD= Standard Deviations

Table 2. Group means (standard deviations) of the IMQ domains, including the subdivision of the HE group into transient and recurrent HE.

| **Assessment** | **HE Group**  ***n=*228**  *Mean* (SD) | **Anxious/ depressed**  **Group**  ***n*=71**  *Mean* (SD) | **Transient HE***  ***n=*114**  *Mean* (SD) | **Recurrent HE***  ***n*=42**  *Mean* (SD) | **Controls**  ***n=*802**  *Mean* (SD) |
| --- | --- | --- | --- | --- | --- |
| **Age 1** |  |  |  |  |  |
| Communication | 39.8 (1.5) | 41.7 (1.3) | 41.5 (1.5) | 37.5 (1.4) | 42.3 (1.4) |
| Gross Motor | 53.2 (1.2) | 54.6 (0.8) | 55.0 (1.0) | 49.8 (1.4) | 54.3 (1.0) |
| Fine Motor | 52.2 (1.0) | 53.3 (0.8) | 54.2 (0.9) | 49.3 (1.1) | 53.6 (0.8) |
| Adaptive | 46.2 (1.3) | 48.7 (1.1) | 46.7 (1.2) | 46.6 (1.1) | 49.6 (1.1) |
| Personal-Social | 45.6 (1.2) | 45.8 (1.1) | 47.8 (1.1) | 44.9 (1.2) | 47.9 (1.1) |
| **Age 2** |  |  |  |  |  |
| Communication | 48.5 (1.4) | 53.5 (0.8) | 51.5 (1.1) | 43.2 (1.6) | 53.3 (1.0) |
| Gross Motor | 54.9 (0.7) | 54.9 (0.7) | 55.6 (0.6) | 53.9 (0.7) | 55.9 (0.7) |
| Fine Motor | 51.5 (0.7) | 51.6 (1.0) | 53.6 (0.7) | 46.5 (0.9) | 53.1 (0.7) |
| Adaptive | 47.4 (0.9) | 48.4 (1.1) | 48.7 (1.0) | 46.3 (1.3) | 51.0 (0.9) |
| Personal-Social | 47.3 (0.8) | 47.9 (0.8) | 48.1 (0.7) | 45.2 (1.0) | 48.6 (0.8) |
| **Age 3** |  |  |  |  |  |
| Communication | 49.9 (1.1) | 52.0 (1.0) | 51.6 (0.9) | 44.4 (1.4) | 53.0 (0.9) |
| Gross Motor | 53.5 (1.0) | 52.5 (1.0) | 54.5 (0.8) | 49.7 (1.2) | 55.5 (0.7) |
| Fine Motor | 48.9 (1.3) | 49.3 (1.4) | 48.9 (1.2) | 48.1 (1.3) | 52.2 (1.1) |
| Adaptive | 48.8 (1.3) | 50.9 (1.3) | 50.3 (1.1) | 46.6 (1.5) | 53.2 (1.1) |
| Personal-Social | 45.8 (1.1) | 45.9 (0.9) | 46.4 (1.0) | 43.9 (1.2) | 47.8 (0.9) |

*Note:* *subgroups of the HE group

Table 3. IMQ cut-off scores (Maximum score for each=60)

| **Assessment** | **Cut-off score** |
| --- | --- |
| **Age 1** |  |
| Communication | 15.8 |
| Gross Motor | 18 |
| Fine Motor | 28.4 |
| Adaptive | 25.2 |
| Personal-Social | 20.1 |
| **Age 2** |  |
| Communication | 36.5 |
| Gross Motor | 36 |
| Fine Motor | 36.4 |
| Adaptive | 32.9 |
| Personal-Social | 35.6 |
| **Age 3** |  |
| Communication | 38.7 |
| Gross Motor | 35.7 |
| Fine Motor | 30.7 |
| Adaptive | 38.6 |
| Personal-Social | 38.7 |

Table 4. Overall and age specific developmental scores between ages 1 and 3 and risk of later HE (also subdivided into Transient or Recurrent) and Anxiety/Depression (compared with Controls (*n*=802) (reference group is Controls) by gender (Males)

|  | **Communication** | **Gross Motor** | **Fine Motor** | **Adaptive** | **Personal**  **-Social** |
| --- | --- | --- | --- | --- | --- |
|  | OR (CI) | OR (CI) | OR (CI) | OR (CI) | OR (CI) |
| **HE group** |  |  |  |  |  |
| Mixed Effect Model | 1.40  (0.62-3.22) | 1.33  (0.61-2.86) | 1.43  (0.15-14.28) | 1.25  (0.76-2.04) | 1.17  (0.68-2.04) |
| Age 1 year | **1.33**  (1.05-1.66) | 1.12  (0.92-1.37) | 1.19  (0.97-1.45) | **1.49**  (1.19-1.85) | **1.26**  (1.02-1.56) |
| Age 2 years | **1.41**  (1.15-1.75) | 1.20  (0.97-1.52) | 1.19  (0.92-1.54) | **1.64**  (1.28-2.04) | 1.20  (0.93-1.54) |
| Age 3 years | **1.32**  (1.08-1.61) | 1.20  (0.98-1.52) | **1.32**  (1.09-1.67) | **1.28**  (1.03-1.56) | 1.22  (0.98-1.54) |
| **Anxious/depressed group** |  |  |  |  |  |
| Mixed Effect Model | 0.99  (0.50-1.92) | 1.20  (0.68-2.12) | 1.07  (0.48-2.38) | 1.23 (0.67-2.28) | 1.23  (0.67-2.28) |
| Age 1 year | 1.04  (0.72-1.52) | 1.03  (0.73-1.47) | 1.14  (0.82-1.59) | 1.22  (0.84-1.75) | 1.32  (0.97-1.85) |
| Age 2 years | 0.87  (0.57-1.30) | 1.19  (0.86-1.64) | 1.08  (0.73-1.61) | 1.33  (0.95-1.89) | 1.06  (0.71-1.59) |
| Age 3 years | 1.03  (0.72-1.45) | **1.36**  (1.01-1.85) | 1.07  (0.77-1.52) | 1.12  (0.81-1.56) | 1.22  (0.85-1.75) |
| **Transient HE group** |  |  |  |  |  |
| Mixed Effect Model | 1.19  (0.65-2.17) | 1.15  (0.66-2.00) | 1.11  (0.63-1.96) | **7.70**  (1.03-62.5) | 1.03  (0.47-2.27) |
| Age 1 year | 1.23  (0.93-1.61) | 0.92  (0.68-1.23) | 1.01  (0.76-1.32) | **1.35**  (1.01-1.82) | 1.12  (0.86-1.47) |
| Age 2 years | 1.21  (0.92-1.61) | 1.05  (0.59-1.45) | 0.89  (0.62-1.27) | **1.47**  (1.09-1.96) | 1.11  (0.81-1.54) |
| Age 3 years | 1.19  (0.93-1.52) | 1.13  (0.86-1.52) | **1.39**  (1.09-1.78) | 1.23  (0.96-1.6) | 1.16  (0.88-1.54) |
| **Recurrent HE group** |  |  |  |  |  |
| Mixed Effect Model | 2.77  (0.67-11.1) | 1.41  (0.79-2.50) | 1.75  (0.62-5.00) | 1.45  (0.51-4.17) | 1.64  (0.50-5.26) |
| Age 1 year | 1.44  (0.67-2.38) | **1.52**  (1.05-2.13) | **1.49**  (1.02-2.17) | 1.40  (0.87-2.27) | 1.45  (0.95-2.22) |
| Age 2 years | **2.43**  (1.49-3.84) | 1.32  (0.82-2.13) | **2.04**  (1.14-3.70) | **1.70**  (1.06-2.70) | 1.32  (0.75-2.27) |
| Age 3 years | **1.82**  (1.30-2.56) | **1.61**  (1.05-2.44) | 1.16  (0.71-1.89) | 1.25  (0.80-1.96) | 1.49  (0.92-2.44) |

*Note*: OR, Odds Ratio, CI, Confidence Intervals. Bolded text represents analyses where 95% CI do not cross 1.

Odds ratios are adjusted for sex at birth, age, birthweight, maternal highest qualification and family income at each year.

Table 5. Overall and age specific developmental scores between ages 1 and 3 and risk of later HE (also subdivided into Transient or Recurrent) and Anxiety/Depression (compared with Controls (*n*=802) (reference group is Controls) by gender (females)

|  | **Communication** | **Gross Motor** | **Fine Motor** | **Adaptive** | **Personal**  **-Social** |
| --- | --- | --- | --- | --- | --- |
|  | OR (CI) | OR (CI) | OR (CI) | OR (CI) | OR (CI) |
| **HE group** |  |  |  |  |  |
| Mixed Effect Model | 1.13  (0.65-1.96) | 1.09  (0.56-2.08) | 1.09  (0.53-2.22) | 1.12  (0.69-1.82) | 1.09  (0.63-1.89) |
| Age 1 year | 0.93  (0.7-1.21) | 1.02  (0.81-1.30) | 0.96  (0.75-1.23) | 1.08  (0.86-1.37) | 0.90  (0.69-1.18) |
| Age 2 years | **1.37**  (1.03-1.82) | 1.12  (0.86-1.47) | **1.30**  (1.01-1.64) | 1.16  (0.90-1.52) | 1.22  (0.93-1.61) |
| Age 3 years | **1.33**  (1.04-1.79) | **1.33**  (1.04-1.72) | **1.30**  (1.01-1.67) | **1.52**  (1.19-1.89) | 1.20  (0.94-1.53) |
| **Anxious/depressed group** |  |  |  |  |  |
| Mixed Effect Model | 1.01  (0.50-2.04) | 1.15  (0.62-2.13) | 1.15  (0.60-2.22) | 1.11 (0.60-2.04) | 1.09  (0.56-2.13) |
| Age 1 year | 1.01  (0.64-1.56) | 0.74  (0.39-1.40) | 0.79  (0.46-1.39) | 0.80  (0.50-1.27) | 1.01  (0.65-1.56) |
| Age 2 years | 1.05  (0.57-1.96) | 1.22  (0.78-1.89) | 1.37  (0.93-1.59) | 1.26  (0.81-1.96) | 1.15  (0.71-1.85) |
| Age 3 years | 1.08  (0.68-1.75) | 1.36  (0.95-2.00) | 1.32  (0.88-1.92) | 1.12  (0.75-1.64) | 1.17  (0.75-1.85) |
| **Transient HE group** |  |  |  |  |  |
| Mixed Effect Model | 0.92  (0.41-2.04) | 1.05  (0.58-1.92) | 1.02  (0.46-2.27) | 1.12  (0.45-2.78) | 0.93  (0.42-2.08) |
| Age 1 year | 0.84  (0.57-1.22) | 0.96  (0.68-1.40) | 0.66  (0.42-1.06) | 1.35  (1.00-1.82) | 0.80  (0.53-1.19) |
| Age 2 years | 0.90  (0.55-1.47) | 1.07  (0.76-1.52) | 1.06  (0.74-1.54) | 1.04  (0.72-1.52) | 1.05  (0.64-1.41) |
| Age 3 years | 1.06  (0.72-1.56) | 1.22  (0.86-1.69) | 1.22  (0.87-1.78) | **1.39**  (1.01-1.89) | 1.11  (0.80-1.59) |
| **Recurrent HE group** |  |  |  |  |  |
| Mixed Effect Model | 1.82  (0.94-3.57) | 1.52  (0.69-3.33) | 1.70  (0.90-3.13) | 1.41  (0.75-2.63) | 1.45  (0.78-2.70) |
| Age 1 year | 1.39  (0.87-2.22) | 1.41  (0.96-2.04) | 1.44  (0.97-2.17) | 1.02  (0.66-1.56) | 1.12  (0.70-1.78) |
| Age 2 years | **1.85**  (1.08-3.22) | 1.22  (0.71-2.08) | **2.12**  (1.29-3.57) | 1.03  (0.57-1.85) | **2.00**  (1.17-3.45) |
| Age 3 years | **1.82**  (1.20-2.70) | **1.81**  (1.23-2.70) | **1.51**  (1.01-2.33) | **1.61**  (1.11-2.33) | **1.64**  (1.04-1.63) |

*Note*: OR, Odds Ratio, CI, Confidence Intervals. Bolded text represents analyses where 95% CI do not cross 1.

Odds ratios are adjusted for sex at birth, age, birthweight, maternal highest qualification and family income at each year.
